# Study on Hepatotoxicity of Benzophenone-3 at Environmental Concentration in Postpartum Mice

**DOI:** 10.3390/toxics13121014

**Published:** 2025-11-22

**Authors:** Huai-Fan Zhai, Ya-Nan Tian, Yu-Xin Sheng, Ya-Jia Pu, Yan-Rong Gao, Jia-Yi Chen, Jia-Di Liu, Jia Ma, Hai-Ming Xu, Peng-Bin Yang, Hong-Mei Li

**Affiliations:** 1 The Key Laboratory of Fertility Preservation and Maintenance of the Ministry of Education, School of Public Health, Ningxia Medical University, Yinchuan 750004, Chinaxuhaiming1986@nxmu.edu.cn (H.-M.X.); 2 School of Clinical Medical, Ningxia Medical University, Yinchuan 750004, China; 3 School of Biological Science & Engineering, North Minzu University, Yinchuan 750021, China

**Keywords:** benzophenone-3, environmentally relevant concentrations, hepatotoxicity, postpartum mice, integrated biomarker response (IBR)

## Abstract

Benzophenone-3 (BP-3), a widely used ultraviolet absorber in various scenarios, exhibits estrogenic toxicity at environmental concentrations—as demonstrated in our prior work. Given the importance of hepatic metabolism and the limitations of previous hepatotoxicity research (high-dose models, lack of mammalian data, etc.), we evaluated BP-3’s hepatic effects on postpartum mice at environmentally relevant levels. Postpartum mice were exposed to BP-3 via drinking water from postpartum day 1 (PPD1) to PPD35. Groups solvent control (0.001% DMSO), 10–1000 nM BP-3, and diethylstilbestrol (DES) were established. Basic growth performance, histopathological changes, and a range of molecular indicators were assessed. The results showed that BP-3 exposure induced dose-dependent increases in liver weight, histopathological alterations (sinusoidal dilation, hepatocyte edema, and necrosis), and significant upregulation of oxidative stress markers (*Ros*, *Mda*), chemokines (*Ccl27a*/*b*), and inflammatory factors (*Tnf-α*, *Il-6*, *Nf-κb*) at the mRNA level (all *p* < 0.05). Conversely, levels of antioxidant enzymes (*Cat*, *Sod1/2*) and anti-inflammatory factor *Ho-1* were markedly decreased (*p* < 0.05). A clear dose-effect relationship was confirmed using the Integrated Biomarker Response (IBR) framework. This pioneering study establishes the hepatotoxicity of environmentally relevant BP-3 levels in mammals and offers methodological insights for endocrine disruptor assessment.

## 1. Introduction

As living standards rise and awareness of health issues like skin cancer increases, daily sun care is garnering increasing attention. Thus, benzophenones (BPs)—organic UV absorbers—are increasingly used in sunscreens and skincare products. Benzophenone-3 (BP-3) is particularly popular in sunscreens due to its effective UV absorption. BP-3 is also utilized in plastics, paints, and adhesives to protect these materials from sun-induced aging and degradation [1,2,3]. Widespread BP-3 usage has led to increasing environmental levels [4,5,6]. It has been detected in various water sources, including drinking, tap, river, and seawater [7], with concentrations up to 230 μg/L in river water [8], and 295 ng/L in tap water [9]. Despite wastewater treatment, BP-3 persists in the environment, accumulating in various water bodies, sediments, and air [10,11,12]. BP-3 degrades slowly, with a degradation rate of 4% in water over 28 days and a surface water half-life of several weeks, which is 7–9 times longer in winter than in summer [13]. BP-3 has also been found in human plasma (0.07–13.56 ng/mL) [14], urine (0.68–6740 ng/mL) [3], breast milk (0.5–72.4 ng/mL) [15], and adipose tissue (<LOD–1.48 ng/g) [16]. It was even found in cod livers from Oslo, Norway at levels up to 1037 ng/g dry weight (dw) [17].

BP-3 has been shown to exhibit estrogenic, anti-androgenic, acute, genotoxic, and neurotoxic effects [2,3]. A study found that UV filters, including BP-3, tended to accumulate in the liver, where BP-3 undergoes biotransformation following ingestion [18]. Both BP-3 and its metabolites can be toxic to the liver. As for aquatic animal experiments, Zhang et al. observed that clown anemonefish hepatocytes showed alterations and accumulated lipid droplets after exposure to 10 or 50 μg/L BP-3 for 7–14 days, with abnormal lipase and antioxidant enzyme activities [19]. Fourteen-day exposure to 50 mg/L BP-3 significantly disrupted phenylalanine and tyrosine metabolism in *Sparus aurata* liver, accompanied by abnormal lipid metabolism and oxidative stress [20]. Another study found that exposing zebrafish to 44 μg/L BP-3 for 30 or 45 days reduced liver glycogen and triglyceride levels and significantly elevated alanine aminotransferase (ALT) and aspartate aminotransferase (AST) activities. Additionally, superoxide dismutase (SOD), catalase (CAT), glutathione peroxidase (GPX), and glutathione (GSH) activities were notably decreased [21]. Wang et al. found that 14-day exposure to 1 pg/L, 30 μg/L, or 300 μg/L BP-3 in zebrafish disrupted liver metabolism, induced oxidative stress, and caused inflammation [22]. Following three consecutive days of intraperitoneal administration of 3.0 μg/g/day BP-3, *Scophthalmus maximus* showed a significant increase in SOD activity and total glutathione concentration compared to the control group. After seven days of injections, glutathione reductase (GR) activity increased notably, and lipid peroxide (LPO) levels decreased substantially [23]. Liu et al. reported that *Carassius auratus* exposed to 0.5 mg/L BP-3 for 7 days had reduced SOD and CAT activities, increased glutathione S-transferase (GST) activity, and formed ring-like clusters of liver cells compared to controls [24]. These studies suggest BP-3 exposure disrupts fish liver function via inflammation and oxidative stress, though the mechanisms remain poorly understood. Current research typically involves short-term, high-dose exposure—conditions that may not reflect natural environments. Limited data exist on BP-3’s effects on mammalian liver toxicity. Only the U.S. National Toxicology Program (NTP) found that pregnant Sprague Dawley rats exposed to 3000, 10,000, and 30,000 mg/kg BP-3 via diet from gestation day 6 (GD6) to postnatal day 28 (PND28) had increased liver weights in the first filial generation (F1) and hepatomegaly in second filial generation (F2) rats receiving the highest dose. From today’s perspective, the study had limitations, including high exposure levels, a lack of molecular/mechanistic analysis, and no investigation into the effects of environmentally relevant BP-3 concentrations on mammalian hepatotoxicity [25].

Based on the above findings, this study hypothesizes that environmentally relevant concentrations of BP-3 may induce hepatic toxicity in postpartum mice.

Postpartum mice are an ideal and unique animal model for studying environmental estrogen-induced liver toxicity. Their core advantage lies in their ability to accurately simulate the liver metabolic vulnerability of lactating women due to drastic hormone fluctuations, while revealing the “time window sensitive toxicity” mechanism of environmental estrogens [26].

Given the susceptibility of postpartum mice, this study aims to evaluate the hepatic toxicity of BP-3 at environmentally relevant concentrations—focusing on organ, tissue, and molecular-level impacts—and identify key toxic reaction indicators in the liver. We used postpartum ICR female mice to achieve this. The findings are expected to inform future research on the potential liver toxicity of environmental estrogens.

## 2. Materials and Methods

### 2.1. Animal Rearing

The 8-week-old ICR pregnant mice, weighing over 80 g, provided by the Animal Experimental Center of Ningxia Medical University, were housed in individually ventilated cages. The temperature was maintained within a range of 23 ± 1 °C, the humidity level was kept between 40–50%, and a light-dark cycle of 12 h was implemented to ensure an adequate supply of food and water. Mice exhibiting severe illness, accidental death, or abnormally significant weight loss during the acclimation or experimental phase were excluded. All animal experiments conducted in this study were performed in strict compliance with the ARRIVE guidelines. The procedures were in accordance with the U.K. Animals (Scientific Procedures) Act 1986. This study was conducted in strict accordance with the ethical code of animal experiments and was approved by the Ethics Committee of the Animal Experiments Center of Ningxia Medical University on 6 March 2023 (approval number NXMU-2023-G038).

### 2.2. BP-3 Exposure Concentrations

This study assessed BP-3 hepatotoxicity in postpartum mice via drinking water to mimic chronic low-dose exposure and quantify daily intake for toxicological comparisons. Two exposure concentrations were set: low-dose (2.28 μg/L, background environmental levels) and high-dose (228 μg/L, simulating pollution hotspots or long-term exposure), following WHO multi-gradient dosing principles. Pharmacokinetic extrapolation and modeling showed that mice exposed to 206.4 μg/L BP-3 reached blood concentrations equivalent to median human levels (200 ng/L) (Tables S1 and S2). This supports the study’s ecological relevance and alignment with environmental and human exposure levels.

### 2.3. Experiment Design

To investigate the hepatotoxic effects of BP-3 at varying concentrations on pregnant mice, we used a randomized design to assign mice to four groups (*n* = 10; sample size determined by subacute toxicity experiment design principles): a control group treated with DMSO (Solarbio Life Sciences, Beijing, China), a low-dose BP-3 (Shanghai Aladdin Biochemical Technology Co., Ltd., Shanghai, China) group (10 nM), a high-dose BP-3 group (1000 nM), and a positive control group treated with diethylstilbestrol (DES, Shanghai Aladdin Biochemical Technology Co., Ltd., Shanghai, China). To control for potential confounding factors (e.g., treatment order, measurement sequence, cage location), we employed complete randomization to ensure baseline group balance, a Latin square design to counterbalance treatment order, and systematic cage position rotation to mitigate environmental bias. This integrated approach ensured uniform distribution of non-experimental factors across groups, effectively minimizing systematic error. The experiments were repeated three times. The day of parturition was designated as PPD1, and the dams were continuously exposed to BP-3 via drinking water from PPD1 to PPD35. Rigorous double-blinding was implemented throughout the study to prevent bias: an independent staff member handled group allocation, ensuring the research team remained blinded to assignments. Personnel conducting experimental exposures were blinded to group identities, and this blinding extended to statisticians and outcome assessors during data processing and analysis to prevent subjective interpretation. Animals were continuously monitored throughout the experiment, with operational protocols promptly adjusted based on their condition. Per ethical guidelines, procedures were halted immediately if signs of pain or distress occurred, with necessary interventions provided. On PPD35, the dams were euthanized by cervical dislocation, and liver tissues were harvested. To assess the impact of environmentally relevant BP-3 concentrations on the liver morphology of the dams, we examined the liver’s large lobe under a stereomicroscope. Hematoxylin-eosin (HE) staining was conducted to assess liver histopathology (*n* = 3). Furthermore, reverse transcription quantitative polymerase chain reaction (RT-qPCR) was used to evaluate the influence of BP-3 on hepatic oxidative stress and the expression of antioxidant factors (*n* = 6). By integrating the outcomes of immunofluorescence (IF) and antioxidant enzyme levels (*n* = 3), we calculated the integrated biomarker response (IBR) index, which reflects the hepatic effects of BP-3 exposure in the dams in relation to the overall biomarker expression levels. The chemical information and main reagents are shown in Tables S3 and S4. During data analysis, all animals that completed the exposure experiment and provided valid data were included.

### 2.4. Morphology and Histopathology for Liver

Mouse liver lobules (*n* = 3) were fixed in 4% paraformaldehyde (4% PFA) for 18 to 24 h and subsequently photographed under a stereo microscope MZ10 F (Leica Microsystems, Wetzlar, Germany) to observe their morphology and size. Subsequent to fixation, the livers underwent a dehydration process, followed by paraffin embedding, and were sliced into 5 μm sections. These sections were then baked in a thermostatic oven at 60 °C for 2 to 3 h, stained, and sealed with neutral resin. The structural modifications in the liver samples were meticulously examined under a fluorescence microscope DM2000 (Leica Microsystems, Wetzlar, Germany). During quantitative analysis, three samples were selected from each group, and 3–4 sections were chosen from each sample. Ultimately, ten fields of view (400 × 300 μm) were captured per group. The sinusoidal diameter (transverse diameter) was measured using Fiji Image J 1.35 software. Using the sinusoidal diameter of the CON group as the standard, values greater than this were defined as dilation. Based on this, the sinusoidal diameter and the number of dilated sinusoids were quantified, alongside the counting of necrotic cells.

### 2.5. RT-qPCR

Liver tissue samples (*n* = 6), measuring approximately 10 mg, were obtained by means of excision, after which the lysate was added for homogenization. Subsequently, total RNA extraction was performed using an AFTSpin Tissue/Cell Fast RNA Extraction Kit for Animal (ABclonal Technology, Wuhan, China). A NanoDrop 2000 (Gene Company Limited, Hong Kong, China) microspectrophotometer was employed to determine the concentration of total RNA extracted. Using the ABScript Ⅲ RT Master Mix for qPCR with gDNA Remover kit (ABclonal Technology, Wuhan, China) to reverse transcribe RNA into cDNA. This was followed by amplification using the 2X Universal SYBR Green Fast qPCR Mix kit (ABclonal Technology, Wuhan, China). Gapdh was utilized as the internal control to ensure the accuracy of the target gene content in the samples. The expression levels of oxidative stress markers *Ros*, *Mda*, chemokines *Ccl27a*, *Ccl27b*, inflammatory factors *Nf-kb*, *Il-6*, *Tnf-α*, antioxidant enzymes *Sod1*, *Sod2*, *Cat*, antioxidant factors *Ho-1*, *Nrf2*, estrogen receptors *Esr1*, *Esr2* were determined using the 2-ΔΔCt method. The detailed reagents and primers utilized in this experiment are enumerated in Tables S5 and S6.

### 2.6. Immunofluorescence (IF)

The large lobe of the liver (*n* = 3) was fixed with 4% PFA, followed by dehydration processing, then embedded in paraffin and sectioned into 5 μm sections. These sections were subsequently baked in a constant temperature oven at 60 °C for 2–3 h. Sections underwent dewaxing in xylene, hydration in gradient alcohol, and rinsing for 2 min. High-pressure antigen repair was followed by PBS (Wuhan Servicebio Technology Co., Ltd., Wuhan, China) washing and incubating with goat serum (Beyotime Biotechnology, Shanghai, China) at 4 °C for 40 min. Primary antibody incubation occurred overnight at 4 °C. The following day, the slices were permitted to return to ambient temperature, washed with PBS, and incubated with the secondary antibody for 1 h in conditions devoid of light. After another PBS wash, DAPI (Shandong Sparkjade Biotechnology Co., Ltd., Jinan, China)-sealed slides were observed under a fluorescence microscope DM2000 (Leica Microsystems, Wetzlar, Germany). Two negative controls were used: one without the primary antibody and another with non-immune Ig (Figure S1). The detailed reagents are documented in Table S7.

### 2.7. Antioxidant Enzyme Level Detection

The liver tissue (*n* = 3) was homogenized and subsequently centrifuged at 4 °C (10,000 rcf for 10 min), after which the supernatant was harvested for the detection of antioxidant enzyme levels. The activities of catalase (CAT), superoxide dismutase (SOD) and total antioxidant capacity (T-AOC) were determined using the ammonium molybdate method, micromethod, and FRAP method, respectively.

### 2.8. Integrated Biomarker Response, IBR

The Biomarker Composite Response Index (IBR) was first developed by Beliaeff and Burgeot in 2002 to assess the toxicological effects of a particular pollutant on an organism [27]. The steps for IBR calculation are as follows: (1) Calculate the mean and standard deviation of the dataset. (2) Standardize the data using the formula *Y* = (*X* − *m*)/*s*, where *Y* represents the standardized data, *X* denotes the raw biomarker response values, m is the mean, and s is the standard deviation. (3) Assign *Z* based on biomarker activation status: if the biomarker is activated by contamination, set *Z* = *Y*; otherwise, set *Z* = −*Y*. Calculate |*Min*|, where *Min* is the minimum value of the standardized data *Z*. (4) Compute the S-value using *S* = *Z* + |*Min*|. (5) Calculate the star plot area (Integrated Biomarker Response, IBR). Let *n* be the number of selected biomarkers, then *A_i_* = [*S_i_* × (*S_i_* + 1) × Sin (2Π/*n*)]/2, IBR = $\sum_{i=1}^{n}A_{i}$. The IBR index is the sum of standardized biomarker expression or activity changes in each treatment group. Through this index, the toxic effects of pollutants on organisms can be quantitatively assessed. The greater the IBR index, the greater the change in the overall expression or activity of the biomarker compared to the control group, that is, the greater the impact of pollutant exposure on the organism.

### 2.9. Data Analysis

The data were analyzed using IBM SPSS Statistics 27.0 software. The quantitative variables were presented as the mean ± standard deviation (SD). The relative gene expression data of the RT-qPCR assay was presented as the mean ± standard error (SE). Statistical comparisons were first conducted through one-way analysis of variance (ANOVA), with subsequent multiple comparisons being performed using either the LSD-*t* test or Dunnett’s procedure. When *p* < 0.05, a statistically significant difference was identified.

## 3. Results

### 3.1. BP-3 Exposure Induced Liver Morphological Changes in Postpartum Mice (PPD1-PPD35)

**Figure 1:**
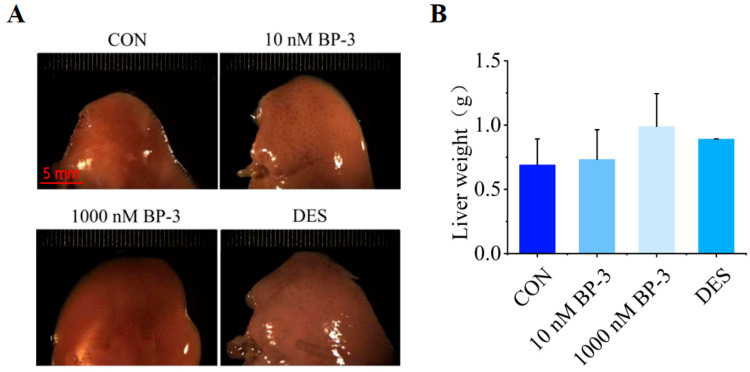
The effects of postpartum mice exposed to 10 and 1000 nM BP-3 for 35 days on liver morphology (**A**) and weight of liver lobes (**B**). Data were presented as the mean SD. CON: control group. Scale bar: 5 mm.

Under the stereomicroscope, compared with the control group, the volume of liver lobes in 10 nM BP-3 treatment group and 1000 nM BP-3 treatment group increased in turn (Figure 1A). The results of ANOVA showed that the liver weight of the PPD 35 postpartum mice in the BP-3 exposure group was not significantly different from that in the control group, but the liver weight showed an overall upward trend (Figure 1B).

### 3.2. BP-3 Exposure Caused Liver Structure Damage in Postpartum Mice (PPD1-PPD35)

**Figure 2:**
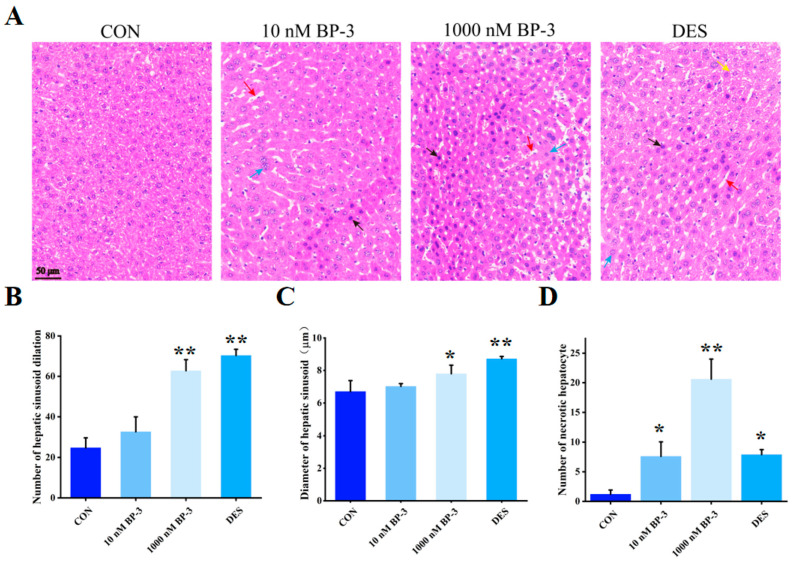
The effects of postpartum mice exposed to 10 and 1000 nM BP-3 for 35 days on liver tissue structure (**A**), Diameter of hepatic sinusoid (**B**), relative numbers of hepatic sinusoid (**C**), relative numbers of necrotic cell (**D**). Different arrows represent different lesions. Red represents dilated hepatic sinusoids, blue represents hepatocyte edema, black represents necrotic hepatocytes, and yellow represents hepatocyte steatosis. Data were presented as the mean SD. * represents significant differences from the control group (*p* < 0.05) and ** represents *p* < 0.01. CON: control group. 5 μm cross section. Scale bar: 50 μm.

The hepatocytes of the control group exhibited a compact arrangement and uniform staining. The nuclei were found to be round in shape, with occasional instances of double nuclei. In the DES group, hepatocytes exhibited marked signs of edema. Fatty degeneration occurred in some cells, and there were many fat vacuoles in the cells, hepatic sinusoid dilation, some hepatocytes were necrotic. Compared with the control group, hepatic sinusoid dilation was observed in the liver tissue of the postpartum mice exposed to 10 nM BP-3. Hepatocytes generally showed mild edema and increased volume, there were a small number of scattered necrotic cells, nuclear pyknosis, dense cytoplasmic staining, acidophilic enhancement. The expansion of hepatic sinusoid was more obvious in the liver tissue of postpartum mice exposed to 1000 nM BP-3. The number of necrotic hepatocytes increased significantly and they were interconnected in sheets (Figure 2A). Quantitative analysis showed that compared with the control group, the number of dilated hepatic sinusoids in the liver of the 1000 nM BP-3 exposure group was significantly increased (Figure 2B). In comparison with the control group, the high-dose group demonstrated a significant increase in hepatic sinusoid dilation (*p* < 0.05) (Figure 2C). The number of necrotic hepatocytes in the low and high dose BP-3 treatment groups was also significantly higher than that in the control group (Figure 2D).

### 3.3. BP-3 Exposure Caused Oxidative and Inflammation Damage at mRNA Levels in Postpartum Mice (PPD1-PPD35)

**Figure 3:**
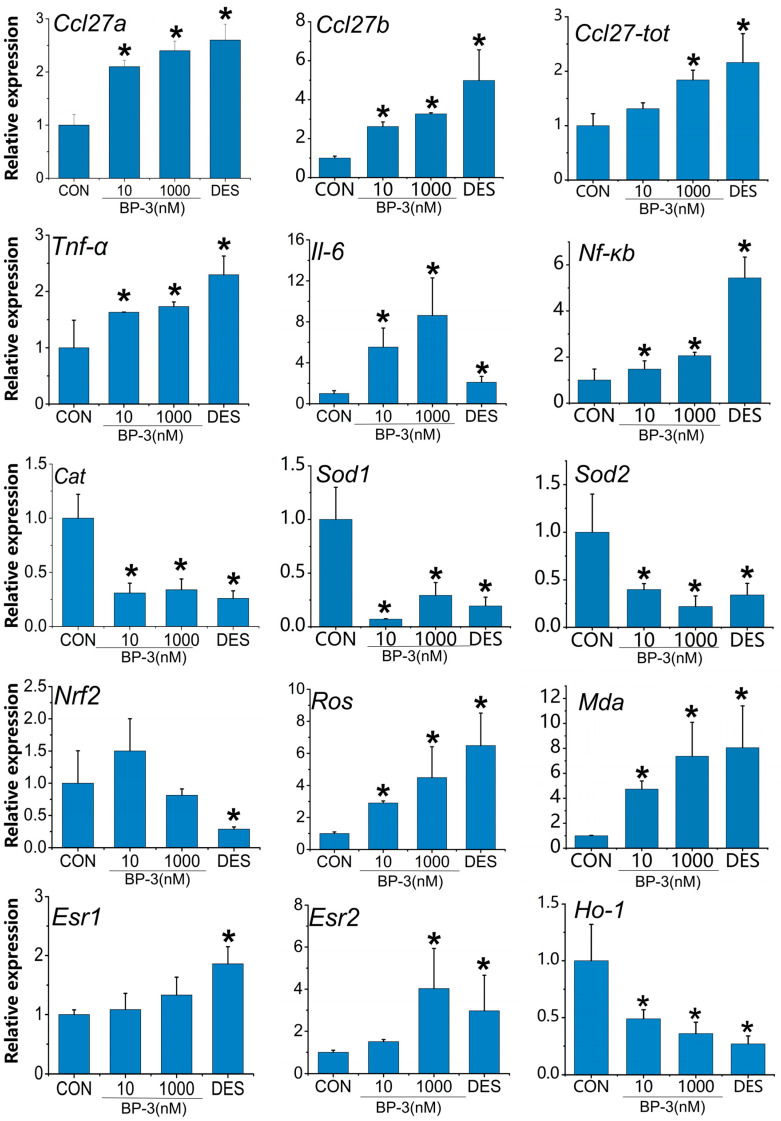
The effects of postpartum mice exposed to 10 and 1000 nM BP-3 for 35 days on relative expression of *Ros*, *Mda*, chemokines (*Ccl27a*, *Ccl27b*), inflammatory factor (*Tnf-α*, *Il-6*, *Nf-κb*), antioxidant enzyme (*Cat*, *Sod1*, *Sod2*), anti-inflammatory factor (*Ho-1*, *Nrf2*). Data were presented as the mean SD. * represents significant differences from the control group (*p* < 0.05). CON: control group.

RT-qPCR was used to detect the mRNA levels of oxidative stress markers (*Ros*, *Mda*), chemokines (*Ccl27a*, *Ccl27b*), inflammatory factors (*Tnf-α*, *Il-6*, *Nf-κb*), antioxidant enzymes (*Cat*, *Sod1*, *Sod2*) and anti-inflammatory factors (*Ho-1*, *Nrf2*) in the liver of postpartum mice exposed to low and high doses of BP-3 for 35 consecutive days. Compared with the control group, the mRNA levels of oxidative stress markers (*Ros*, *Mda*), chemokines (*Ccl27a*, *Ccl27b*), and inflammatory factors (*Tnf-α*, *Il-6*, *Nf-κb*) were significantly increased in the 10 nM and 1000 nM BP-3 exposure groups (*p* < 0.05) (Figure 3). The mRNA levels of antioxidant enzymes (*Cat*, *Sod1*, *Sod2*) and anti-inflammatory factor *Ho-1* were significantly decreased in the low and high dose BP-3 exposure groups, with statistical significance (*p* < 0.05) (Figure 3).

### 3.4. BP-3 Exposure Induced Oxidative and Inflammation Damage at Protein Levels in the Liver of Postpartum Mice (PPD1-PPD35)

**Figure 4:**
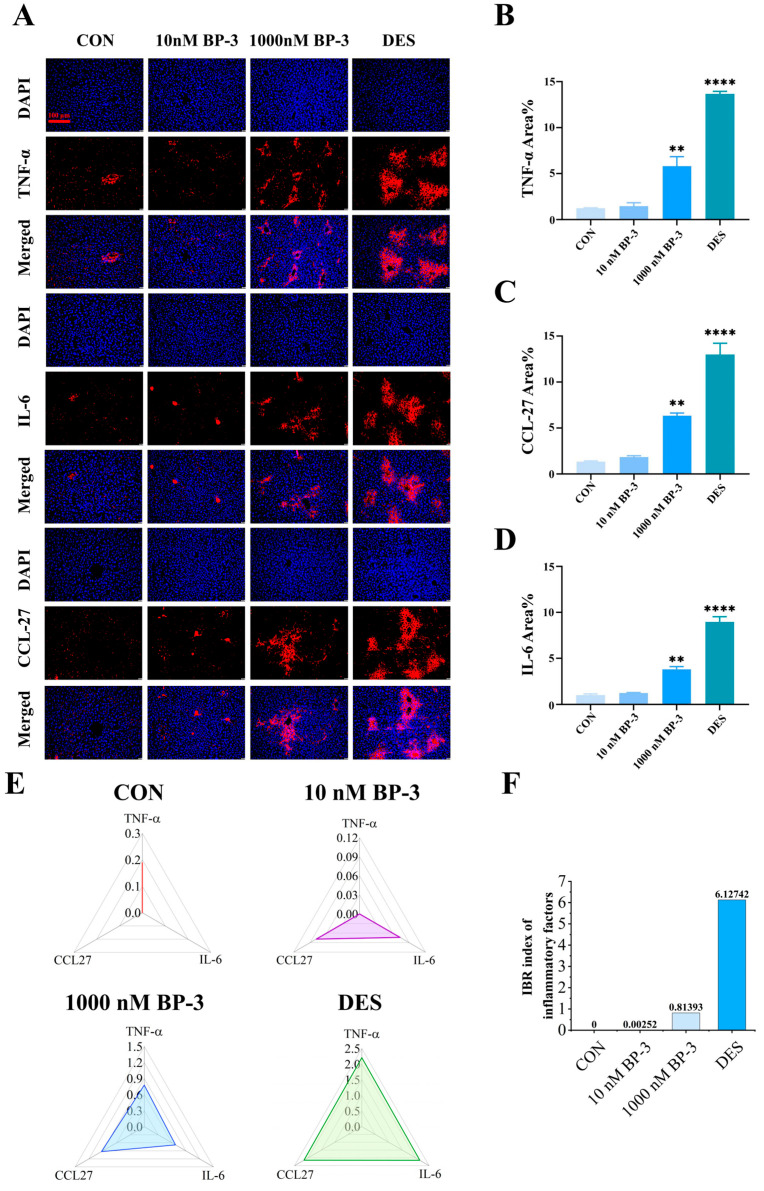
The effects of postpartum mice exposed to 10 and 1000 nM BP-3 for 35 days on hepatic inflammation (**A**), relative fluorescence area of TNF-α (**B**), relative fluorescence area of IL-6 (**C**), relative fluorescence area of CCL27 (**D**), the overall expression level of TNF-α, IL-6 and CCL27 biomarkers (**E**), and the IBR index of inflammatory factor expression (**F**). Data were presented as the mean SD. ** represents significant differences from the control group (*p* < 0.01) and **** represents *p* < 0.0001. CON: control group. 5 μm cross section. Scale bar: 100 μm.

The levels of TNF-α, IL-6 and CCL27 proteins in the livers of postpartum mice were analyzed by IF. The results showed that BP-3 exposure caused obvious inflammatory reaction in the liver of postpartum mice, and the damage around the central vein was the most serious (Figure 4A). The blue fluorescence observed is indicative of the nucleus, which has been stained by DAPI, while the red fluorescence corresponds to the labeled TNF-α, IL-6 and CCL27, respectively. The red fluorescence of the control group was less and dispersed, indicating that the expression of the three proteins in the liver tissue of the mice in this group was low and evenly distributed. The red fluorescence area of the DES group was large and concentrated, indicating that the three proteins were expressed in large quantities and were mostly concentrated in the liver cells around the central vein (Figure 4A). Compared with the control group, the expression level of TNF-α protein in the liver of postpartum mice in the BP-3 exposure group showed an upward trend, and the TNF-α level in the 1000 nM BP-3 group increased significantly (*p* < 0.05) (Figure 4B). In contrast with the control group, the protein levels of IL-6 and CCL27 were also notably higher in the 1000 nM BP-3 group (*p* < 0.05) (Figure 4C,D). As shown in Figure 4E,F, the IBR index calculated based on immunofluorescence data revealed that BP-3 exposure altered the overall expression levels of TNF-α, IL-6, and CCL27. The calculated IBR index was 0 in the control group and approximately 6.127 in the DES group. Compared to the control, the low- and high-dose BP-3 exposure groups exhibited IBR indices of 0.003 and 0.814, respectively, demonstrating a discernible dose-effect relationship. These results suggest that BP-3 exposure induces inflammatory liver injury in postpartum mice.

### 3.5. BP-3 Exposure Changed the Activities of CAT, SOD and T-AOC in the Liver of Postpartum Mice (PPD1-PPD35)

**Figure 5:**
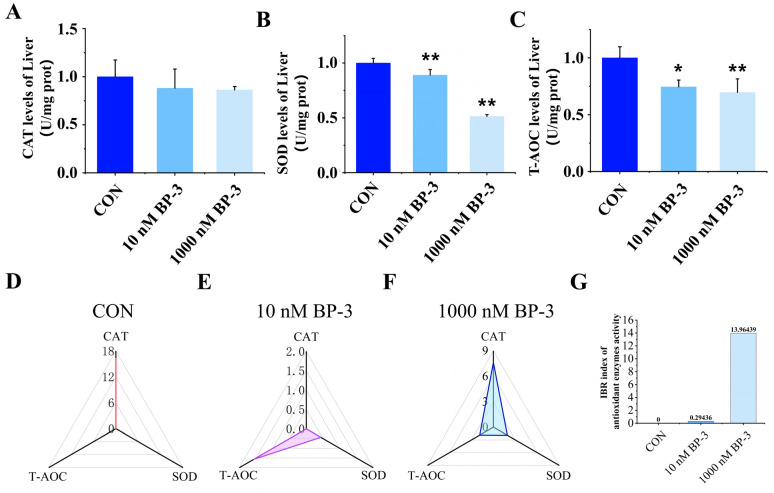
The effects of postpartum mice exposed to 10 and 1000 nM BP-3 for 35 days on the activity of CAT (**A**), SOD (**B**) and T-AOC (**C**) in liver, the overall activity of CAT, SOD and T-AOC in the CON group (**D**), 10 nM BP-3 group (**E**) and 1000 nM BP-3 group (**F**) and the IBR index of antioxidant enzyme activity (**G**). Data were presented as the mean SD. * represents significant differences from the control group (*p* < 0.05) and ** represents *p* < 0.01. CON: control group.

As demonstrated in Figure 5, compared to the control group, although no statistically significant differences were observed in CAT activity following low- and high-dose BP-3 treatments, an overall downward trend was evident (Figure 5A). In contrast, SOD activity in the livers of postpartum mice exposed to 10 nM and 1000 nM BP-3 was significantly reduced relative to the control group (*p* < 0.05) (Figure 5B). Furthermore, T-AOC exhibited significant downward trends in both exposure groups, with these effects displaying dose-dependent relationships (*p* < 0.05) (Figure 5C). To assess the overall impact of BP-3 exposure on hepatic antioxidant capacity, the IBR index was recalculated using normalized data from CAT, SOD, and T-AOC activities (Figure 5D–F). The results demonstrated an IBR index of 0 in the control group. In comparison, the 10 nM and 1000 nM BP-3 exposure groups exhibited IBR indices of 0.294 and 13.964, respectively, indicating significant suppression of hepatic antioxidant capacity following BP-3 exposure, with a clear dose-effect relationship observed (Figure 5G).

## 4. Discussion

BP-3 is widely used for its excellent UV-absorbing performance, inevitably causing environmental pollution and posing health risks. Its prevalence in diverse environmental matrices is well documented. Vila et al. reported BP-3 concentrations up to 10 μg/L in seven Spanish swimming pools [28]. In Iran’s Abeedard hot springs, BP-3 reached 1000 ng/L [29]. In the course of a study of drinking water samples collected from Water Resources Management Area No.13 in southeastern Brazil, BP-3 concentrations were detected at levels as high as 105 ng/L [30]. Owing to its lipophilic properties, BP-3 readily accumulates in humans and has been identified in multiple biological matrices. The maximum serum concentrations detected in Frederiksen ’s study were 5.18 ng/mL, as measured in a group of 300 Danish young males [31]. Breast milk samples from 79 Catalan lactating women showed peak BP-3 levels of 779.9 ng/g [32]. Although BP-3’s diverse toxic effects have been extensively studied [33,34,35,36,37] and preliminary evidence suggests hepatotoxic potential, current research is limited by environmentally irrelevant high doses and a lack of mammalian models—hindering accurate simulation of real-world exposure and reliable interspecies extrapolation of toxicity. The study’s innovative design addresses these gaps, representing a significant advance.

This study is the first to report the hepatotoxic effects of environmentally relevant BP-3 concentrations in postpartum mice. Results demonstrated that BP-3 exposure induced hepatic morphological alterations, characterized by enlarged lobular volume and increased liver weight compared to controls, consistent with NTP-reported liver weight gain in F1 SD rats and hepatomegaly in F2 rats [25]. These findings align with histopathological observations of hepatic congestion and hepatocellular edema. Previous studies showed BP-3 induced lipid droplet accumulation in clownfish hepatocytes [19], triggered apoptosis in zebrafish hepatocytes [22], and elevated ALT/AST levels in zebrafish [21]. However, BP-3-induced changes in this study were more pronounced. Notably, 10 nM BP-3 caused marked hepatocellular edema with cellular enlargement and lighter cytoplasmic staining. Both low- and high-dose BP-3 groups had significantly more necrotic cells than the DES group, whereas hepatic steatosis was only observed in DES-exposed mice. BP-3-exposed groups also showed significantly greater sinusoidal dilation (in severity and frequency) than controls—this dilation may be associated with BP-3’s estrogenic effects [38].

Multiple studies on BP-3-induced hepatotoxicity in aquatic species demonstrate that BP-3 exposure triggers oxidative stress and inflammatory responses in fish livers. Under physiological conditions, organisms maintain redox balance by producing SOD and CAT to scavenge ROS generated during mitochondrial electron transport. However, BP-3-exposed fish livers exhibited significantly reduced activities of oxidative stress biomarkers (SOD, CAT, GPx, and GSH) [20,22], impairing ROS clearance capacity. In this study, BP-3 exposure decreased CAT activity and markedly reduced SOD activity compared to controls, with both low- and high-dose groups showing significantly diminished T-AOC. IBR index calculations revealed dose-dependent impairment of hepatic antioxidant capacity. Furthermore, BP-3 exposure upregulated mRNA levels of *Ros* and *Mda* while downregulating *Sod* and *Cat* transcripts. Consistent with zebrafish studies demonstrating BP-3-induced hepatic inflammation [22], all BP-3-exposed groups exhibited significantly elevated mRNA levels of chemokines (*Ccl27a*, *Ccl27b*) and pro-inflammatory factors (*Tnf-α*, *Il-6*, *Nf-κb*), alongside reduced anti-inflammatory factor *Ho-1* transcripts. Notably, the 1000 nM BP-3 group had substantial increases in TNF-α, IL-6, and CCL27 protein levels, primarily localized around central veins. Immunohistochemistry (IHC)-based IBR index analysis further confirmed robust inflammatory responses in BP-3-exposed mouse livers.

This study differs from previous high-dose BP-3 models by assessing hepatotoxicity at environmentally relevant concentrations via drinking water—enhancing ecological relevance. Notably, it is the first to use postpartum mice as a model: their physiologically vulnerable state and ongoing systemic recovery after childbirth make the liver more susceptible to BP-3-induced stress, enabling more sensitive identification of hepatotoxicity biomarkers. This approach establishes a methodological framework for evaluating hepatic effects of potential endocrine-disrupting chemicals (EDCs).

This study demonstrates the hepatotoxic effects of environmentally relevant BP-3 concentrations in postpartum female mice. Future studies should further elucidate the mechanisms of BP-3-induced hepatotoxicity and explore strategies to protect against liver damage.

## 5. Conclusions

This study demonstrates that environmentally relevant BP-3 exposure induces hepatotoxicity in postpartum mice, as evidenced by significant increases in liver weight, hepatic sinusoid dilation, hepatocellular edema, and necrosis. The observed hepatic damage was associated with oxidative stress and inflammatory responses in liver tissue. As the first demonstration of BP-3’s hepatic toxicity at environmentally relevant levels in postpartum mice, this research underscores potential hepatotoxic risks for mammals—including humans. Moreover, it establishes a methodological foundation for elucidating the hepatotoxicity mechanisms of other endocrine disruptors.

## Data Availability

Data are available from the authors upon request. A detailed research plan was formulated before the start of this study. The research questions (exploring the hepatotoxic effect of benzophenone-3 on postpartum mice), key design features (including animal groupings, BP-3 drinking water exposure dose and cycle, as well as HE staining, RT-qPCR, immunofluorescence and antioxidant enzyme activity detection) were clarified. The downstream detection methods and statistical analysis plan (*t*-test and ANOVA analysis were conducted using SPSS software). This written plan is archived at our research institution. If need this, you can contact the corresponding author.

## Supplementary Materials

The following supporting information can be downloaded at: https://www.mdpi.com/article/10.3390/toxics13121014/s1, Figure S1: Immunofluorescence negative control; Table S1: Descriptive statistics (range and median levels) on global detection (median and maximum concentrations) of benzophenone-3 in human blood samples; Table S2: Design and derivation of BP-3 exposure concentration based on inter-species conversion and pharmacokinetics; Table S3: Chemical information; Table S4: The relevant information of the main reagents; Table S5: Reagents used for RT-qPCR; Table S6: Primer sequences for RT-qPCR; Table S7: Antibodies used for IF staining. (References [2,16,31,39,40,41,42,43,44,45,46,47,48,49,50] are cited in the Supplementary Materials).
