# Supplementary material for: Study on Hepatotoxicity of Benzophenone-3 at Environmental Concentration in Postpartum Mice
Source: Toxics. 2025 Nov 22;13(12):1014. doi: 10.3390/toxics13121014 (PMC12736964; doi:10.3390/toxics13121014)
Supplement: Supplementary file 1 [file toxics-13-01014-s001.zip › toxics-3960867-supplementary.pdf]

toxics

MDPI

Article

Study on Hepatotoxicity of Benzophenone-3 at Environmental Concentration in Postpartum Mice

Huai-Fan Zhai [1,†], Ya-Nan Tian [2,†], Yu-Xin Sheng [1], Ya-Jia Pu [1], Yan-Rong Gao [1], Jia-Yi Chen [1], Jia-Di Liu [2], Jia Ma [1], Hai-Ming Xu [1], Peng-Bin Yang [3,*] and Hong-Mei Li [1,*]

[1] The Key Laboratory of Fertility Preservation and Maintenance of the Ministry of Education, School of Public Health, Ningxia Medical University, Yinchuan 750004, China; xuhaiming1986@nxmu.edu.cn (H.-M.X.)
[2] School of Clinical Medical, Ningxia Medical University, Yinchuan 750004, China
[3] School of Biological Science & Engineering, North Minzu University, Yinchuan 750021, China
[*] Correspondence: 2024907@nmu.edu.cn (P.-B.Y.); lihongmei@nxmu.edu.cn (H.-M.L.)
[†] These authors contributed equally to this work.

Integrated biomarker response (IBR) index calculation methodology

(1) Calculate the mean and standard deviation of the dataset.
(2) Standardize the data using the formula $Y = (X\text{-}m)/s$, where Y represents the standardized data, X denotes the raw biomarker response values, m is the mean, and s is the standard deviation.
(3) Assign Z based on biomarker activation status: if the biomarker is activated by contamination, set $Z = Y$; otherwise, set $Z = -Y$. Calculate $|Min|$, where Min is the minimum value of the standardized data Z.
(4) Compute the S-value using $S = Z + |Min|$.
(5) Calculate the star plot area (Integrated Biomarker Response, IBR). Let n be the number of selected biomarkers, then $A_i=[S_i\times(S_i+1)\times \text{Sin }(2\Pi/n)]/2$,IBR= $\sum_{i=1}^{n} Ai$.

Table S1. Descriptive statistics (range and median levels) on global detection (median and maximum concentrations) of benzophenone-3 in human blood samples.

	Sample	Median (μg L^{-1})	Maximum (μg L^{-1})	Ref.
Global		n.d.—238.0 (0.2)	n.d.—929.0	(Mao et al.,2022)
China	Serum	n.d.	0.14	(Wang et al.,2019)
	Maternal serum	0.12	0.32	(Song et al.,2020)
	Maternal serum	0.14	25.1	(Song et al.,2020)
	Cord serum	0.07	0.3	(Song et al.,2020)
	Cord serum	0.16	56.6	(Song et al.,2020)
	Cord blood	0.59	2.55	(Zhang et al.,2013)
	Blood	0.52	2.20	(Zhang et al.,2013)
	Blood	0.41	2.30	(Zhang et al.,2013)
	Blood	2.09	3.38	(Zhang et al.,2013)
Denmark	Serum	0.16	5.18	(Frederiksen et al.,2021)
	Maternal serum	n.d	1.0	(Krause et al.,2018)
	Maternal serum	n.d	37.0	(Krause et al.,2018)
	Maternal serum	0.27	10.1	(Krause et al.,2018)
	Maternal serum	n.d.	71.8	(Krause et al.,2018)
	Fatal serum	n.d.	10.1	(Krause et al.,2018)
	Plasma	-	200.0	(Janjua et al.,2004)
	Plasma	53.0	226.0	(Janjua et al.,2008)

	Sample	Median (μg L^{-1})	Maximum (μg L^{-1})	Ref.
	Plasma	60.0	236.0	(Janjua et al.,2008)
	Plasma	108.0	257.0	(Janjua et al.,2008)
	Plasma	47.0	295.0	(Janjua et al.,2008)
	Plasma	-	300.0	(Janjua et al.,2004)
	Plasma	160.0	330.0	(Janjua et al.,2008)
	Plasma	92.0	390.0	(Janjua et al.,2008)
	Plasma	187.0	392.0	(Janjua et al.,2008)
	Plasma	192.0	577.0	(Janjua et al.,2008)
	Plasma	41.0	703.0	(Janjua et al.,2008)
	Plasma	186.0	741.0	(Janjua et al.,2008)
	Plasma	238.0	797.0	(Janjua et al.,2008)
	Plasma	53.0	802.0	(Janjua et al.,2008)
	Menstrual blood	1.2	3.1	(Jimenez-Diaz et al.,2016)
Spain	Serum	n.d.	0.23	(Artacho-Cordon et al.,2017)
	Serum	n.d.	0.7	(Vela-Soria et al.,2014)
	Serum	n.d.	1.2	(Vela-Soria et al.,2014)
	Serum	0.55	21.20	(Salamanca-Fernandez et al.,2020)
	Serum	-	200.0	(Tarazona et al.,2013)
	Serum	-	304.0	(Tarazona et al.,2013)
USA	Pooled sera	n.d.	8.7	(Ye et al.,2012)
	Pooled sera	-	11.3	(Ye et al.,2012)
	Blood	21.14	929.0	(Przybyla et al.,2019)

Note:m n.d.: not detected.
-.:concentrations lower than LOD (limit of detection) or LOQ (limit of quantification). .

Table S2. Design and derivation of BP-3 exposure concentration based on inter-species conversion and pharmacokinetics.

Calculation Steps	Basis of Calculation
1 Calculation of interspecies dose equivalent concentration	Literature review indicates that the median blood concentration of BP-3 in the general population is 200 ng/L. Toxicological extrapolation requires incorporation of interspecies variability (10-fold) and safety factors (3-fold), resulting in a total adjustment factor of 30-fold.<br>The target blood concentration range in mice is adjusted to: 200 *ng/L* × 30 = 6000 *ng/L*, equivalent to human exposure levels within the same order of magnitude.
2 Revision of pharmacokinetic parameters and calculation of exposure concentration	1. Parameter settings and adjustments<br>Target blood concentration (C): 6000 ng/L (6 μg/L)<br>Bioavailability (F): Estimated at 40%–60% (considering first-pass metabolism and solubility limitations), calculated as 50%.<br>Volume of distribution (Vd): 5 L/kg (mouse body weight = 25 g; Vd = 0.125 L).<br>Half-life ($t_{1/2}$): 24 hours (based on physicochemical properties and analog data, theoretical biological half-life of BP-3 in mice is 24–72 hours).<br>2. Clearance and daily intake calculation<br>clearance (CL) = Vd (apparent volume of distribution) × k (depuration rate constant), where k = 0.693/$t_{1/2}$ (elumination half life)<br>$CL=\frac{0.693\times Vd}{t_{1/2}}=\frac{0.693\times 0.125L}{24h}\approx 0.0036L/h\ (0.086L/day)$<br>Steady-state daily intake:

$$\text{Dose}=\frac{C\times CL\times\tau}{F}=\frac{6\mu g/L\times0.086L/day\times1}{0.5}\approx 1.032\mu g/day$$

3.Drinking water exposure concentration derivation

Daily water intake in mice: 5mL/day

$$\text{Exposure concentration}=\frac{1032ng/day}{5mL/day}=206.4\mu g/L$$

Table S3. Chemical information.

Chemicals	CAS NO.	Manufacturers	Cat No.	Purity
Benzophenone-3 (BP-3)	131--57-7	Shanghai Aladdin	H109416	99%
Diethylstilbestrol (DES)	56-53-1	Biochemical Technology Co., Ltd.	D109025	98%
Dimethyl sulfoxide (DMSO)	67-68-5	Solarbio Life Sciences	D8370	99.9%

Table S4. The relevant information of the main reagents.

Reagents	Manufacturers	Cat. No.
Hematoxylin-Eosin (HE) Stain Kit	Solarbio Life Sciences	G1120
Tissue-Tek O.C.T. Compound		4583
Glutaraldehyde, 2.5%		P1126
Goat Serum	Beyotime Biotechnology	C0265
Anti-fluorescence quenching tablets (containing DAPI)	Shandong Sparkjade Biotechnology Co., Ltd.	EE0015
PBS	Wuhan Servicebio Technology Co., Ltd.	G4202
DPBS		G4201
Universal antibody dilution buffer	SEVEN Bioteon	SW161-02
RNase-free water	Beijing Biotopped Technology Co., LTD	Top0854

Table S5. Reagents used for RT-qPCR.

Kits	Manufacturers	Cat No.
AFTSpin Tissue/Cell Fast RNA Extraction Kit for Animal	ABclonal Technology	RK30120
ABScript III RT Master Mix for qPCR with gDNA Remover	ABclonal Technology	RK20429
2X Universal SYBR Green Fast qPCR Mix	ABclonal Technology	RK21203

Table S6. Primer sequences for RT-qPCR.

Gene symbol	Accession No.	Primer sequence (5'–3')	Length
Ccl27a	NM_001048179.2	F: CACATGGAACTGCAGGAGGCC<br>R:TGTAGTACCAGATTTAAACTGGGTACAGTC	144
Ccl27b	NM_001199959.2	F: CACATGGAACTGCAGGAGGCT<br>R: TGTAGTACCAGATTTAAACTGGGTACAGT	86
Il-6	NM_001314054.1	F: CCAAGAGGTGAGTGCTTCCC<br>R: CTGTTGTTCAGACTCTCTCCCT	114
Nrf2	NM_001399226.1	F: TCTTGGAGTAAGTCGAGAAGTGT<br>R: GTTGAAACTGAGCGAAAAAGGC	170
Sod1	NM_011434.2	F: AACCAGTTGTGTTGTCAGGAC<br>R: CCACCATGTTTCTTAGAGTGAGG	140
Sod2	NM_013671.3	F: CAGACCTGCCTTACGACTATGG<br>R: CTCGGTGGCGTTGAGATTGTT	120
Cat	NM_009804.2	F: AGCGACCAGATGAAGCAGTG<br>R: TCCGCTCTCTGTCAAAGTGTG	166
Tnf- α	NM_013693	F: GACGTGGAACTGGCAGAAGAG<br>R: TTGGTGGTTTGTGAGTGTGAG	228
	NM_008689	F: GGAGGCATGTTCGGTAGTGG	135

Gene symbol	Accession No.	Primer sequence (5’–3’)	Length
Nf- κ b		R:CCCTGCGTTGGATTTCGTG	
Ros	NM_011282	F: CTGCCTAACGTCCTGTGTAAC<br>R: CAGAGTTCCAAAACTGACATCCA	104
Mda	NM_001164477	F: AGATCAACACCTGTGGTAACACC<br>R:CTCTAGGGCCTCCACGAACA	107
Esr1	NM_007956	F: CCTCCCGCCTTCTACAGGT<br>R: CACACGGCACAGTAGCGAG	128
Esr2	NM_010157	F: CTGTGCCTCTTCTCACAAGGA<br>R: TGCTCCAAGGGTAGGATGGAC	129
Ho-1	NM_010442	F: AAGCCGAGAATGCTGAGTTCA<br>R: GCCGTGTAGATATGGTACAAGGA	100

Table S7. Antibodies used for IF staining.

Antibody	Host Species	Manufacturer	Cat. No.	Notes	Dilution ratio
CCL27	Rabbit	Shanghai Abmart Co.,Ltd.	T510201	Chemotactic factor	1:100
TNF-α	Rabbit	Shenyang Wanlei Biotechnology Co., Ltd.	WL01896	inflammatory marker	
IL-6	Rabbit		WL02841	inflammatory marker	
IgG (H&L)	Rabbit	Wuhan Abclonal Biotechnology Co., Ltd.	RS3208	Secondary antibody	

Immunofluorescence negative control

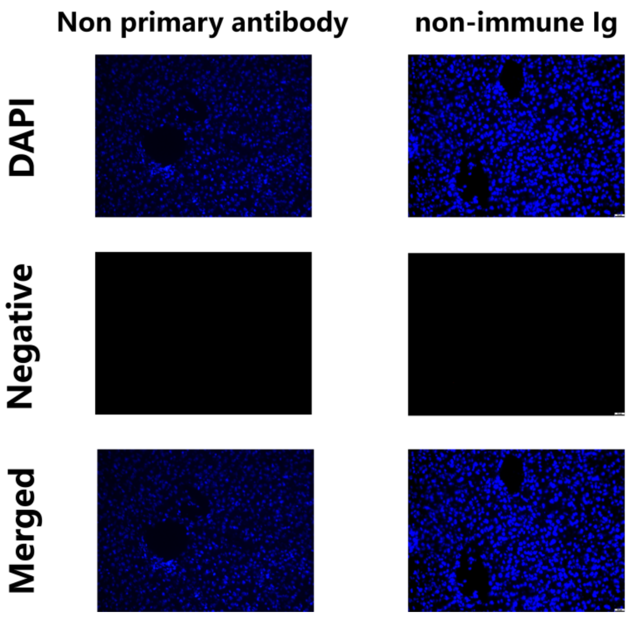

Figure S1. Immunofluorescence negative control. Two negative controls were used: one without the primary antibody and another with non-immune Ig.

Artacho-Cordon, F. , Arrebola, J. P. , Nielsen, O. , Hernandez, P. , Skakkebaek, N. E. , Fernandez, M. F. , Andersson, A. M. , Olea, N. , & Frederiksen, H. Assumed non-persistent environmental chemicals in human adipose tissue; matrix stability and correlation with levels measured in urine and serum. *Environ Res*, 2017, 156: 120-127.

Frederiksen, H. , Krause, M. , Jorgensen, N. , Rehfeld, A. , Skakkebaek, N. E. , & Andersson, A. M. UV filters in matched seminal fluid-, urine-, and serum samples from young men. *J Expo Sci Environ Epidemiol*, 2021, 31(2): 345-355.

Janjua, N. R. , Kongshoj, B. , Andersson, A. M. , & Wulf, H. C. Sunscreens in human plasma and urine after repeated whole-body topical application. *J Eur Acad Dermatol Venereol*, 2008, 22(4): 456-461.

Janjua, N. R. , Mogensen, B. , Andersson, A. M. , Petersen, J. H. , Henriksen, M. , Skakkebaek, N. E. , & Wulf, H. C. Systemic absorption of the sunscreens benzophenone-3, octyl-methoxycinnamate, and 3-(4-methyl-benzylidene) camphor after whole-body topical application and reproductive hormone levels in humans. *J Invest Dermatol*, 2004, 123(1): 57-61.

Jimenez-Diaz, I. , Iribarne-Duran, L. M. , Ocon, O. , Salamanca, E. , Fernandez, M. F. , Olea, N. , & Barranco, E. Determination of personal care products -benzophenones and parabens- in human menstrual blood. *J Chromatogr B Analyt Technol Biomed Life Sci*, 2016, 1035: 57-66.

Krause, M. , Frederiksen, H. , Sundberg, K. , Jorgensen, F. S. , Jensen, L. N. , Norgaard, P. , Jorgensen, C. , Ertberg, P. , Juul, A. , Drzewiecki, K. T. , Skakkebaek, N. E. , & Andersson, A. M. Presence of benzophenones commonly used as UV filters and absorbers in paired maternal and fetal samples. *Environ Int*, 2018, 110: 51-60.

Mao, J. F. , Li, W. , Ong, C. N. , He, Y. , Jong, M. C. , & Gin, K. Y. Assessment of human exposure to benzophenone-type UV filters: A review. *Environ Int*, 2022, 167: 107405.

Przybyla, J. , Kile, M. , & Smit, E. Description of exposure profiles for seven environmental chemicals in a US population using recursive partition mixture modeling (RPMM). *J Expo Sci Environ Epidemiol*, 2019, 29(1): 61-70.

Salamanca-Fernandez, E. , Iribarne-Duran, L. M. , Rodriguez-Barranco, M. , Vela-Soria, F. , Olea, N. , Sanchez-Perez, M. J. , & Arrebola, J. P. Historical exposure to non-persistent environmental pollutants and risk of type 2 diabetes in a Spanish sub-cohort from the European Prospective Investigation into Cancer and Nutrition study. *Environ Res*, 2020, 185: 109383.

Song, S. , He, Y. , Huang, Y. , Huang, X. , Guo, Y. , Zhu, H. , Kannan, K. , & Zhang, T. Occurrence and transfer of benzophenone-type ultraviolet filters from the pregnant women to fetuses. *Sci Total Environ*, 2020, 726: 138503.

Tarazona, I. , Chisvert, A. , & Salvador, A. Determination of benzophenone-3 and its main metabolites in human serum by dispersive liquid-liquid microextraction followed by liquid chromatography tandem mass spectrometry. *Talanta*, 2013, 116: 388-395.

Vela-Soria, F. , Ballesteros, O. , Zafra-Gomez, A. , Ballesteros, L. , & Navalon, A. A new method for the determination of benzophenone-UV filters in human serum samples by dispersive liquid-liquid microextraction with liquid chromatography-tandem mass spectrometry. *Talanta*, 2014, 121: 97-104.

Wang, Y. , Li, G. , Zhu, Q. , & Liao, C. A multi-residue method for determination of 36 endocrine disrupting chemicals in human serum with a simple extraction procedure in combination of UPLC-MS/MS analysis. *Talanta*, 2019, 205: 120144.

Ye, X. , Zhou, X. , Wong, L. Y. , & Calafat, A. M. Concentrations of bisphenol A and seven other phenols in pooled sera from 3-11 year old children: 2001-2002 National Health and Nutrition Examination Survey. *Environ Sci Technol*, 2012, 46(22): 12664-12671.

Zhang, T. , Sun, H. , Qin, X. , Wu, Q. , Zhang, Y. , Ma, J. , & Kannan, K. Benzophenone-type UV filters in urine and blood from children, adults, and pregnant women in China: partitioning between blood and urine as well as maternal and fetal cord blood. *Sci Total Environ*, 2013, 461-462: 49-55.
